# Inhibition of sphingolipid metabolism in osteosarcoma protects against CD151-mediated tumorigenicity

**DOI:** 10.1186/s13578-022-00900-9

**Published:** 2022-10-08

**Authors:** Hongsheng Wang, Xinmeng Jin, Yangfeng Zhang, Zhuoying Wang, Tao Zhang, Jing Xu, Jiakang Shen, Pengfei Zan, Mengxiong Sun, Chongren Wang, Yingqi Hua, Xiaojun Ma, Wei Sun

**Affiliations:** 1 Department of Orthopedics, Shanghai General Hospital, School of Medicine, Shanghai Jiao Tong University, 100 Haining Road, Shanghai, 200080 China; 2 Shanghai Bone Tumor Institution, Shanghai, China

**Keywords:** Osteosarcoma, Tetraspanin CD151, Sphingolipid metabolism, Target therapy

## Abstract

Osteosarcoma is the most common primary bone tumor, with a poor prognosis owing to the lack of efficient molecular-based targeted therapies. Previous studies have suggested an association between CD151 and distinct consequences in osteosarcoma tumorigenicity. However, the potential of CD151 as a therapeutic target has not yet been sufficiently explored. Here, we performed integrated transcriptomic and metabolomic analyses of osteosarcoma and identified sphingolipid metabolism as the top CD151-regulated pathway. CD151 regulates sphingolipid metabolism primarily through SPTCL1, the first rate-limiting enzyme in sphingolipid biosynthesis. Mechanistically, depletion of CD151 enhanced c-myc polyubiquitination and subsequent degradation. c-myc is vital for the transcriptional activation of SPTLC1. Functionally, sphingolipid synthesis and the SPTLC1 inhibitor, myriocin, significantly suppressed the clonogenic growth of CD151-overexpression cells. Importantly, myriocin selectively restrained CD151-high expression tumor growth in preclinical patient-derived xenograft models. Collectively, these data establish that CD151 is a key mediator of sphingolipid metabolism and provide a new approach to developing novel CD151-based targeted therapies for osteosarcoma.

**Supplementary Information:**

The online version contains supplementary material available at 10.1186/s13578-022-00900-9.

## Introduction

Osteosarcoma is the most common primary bone tumor, occurring in approximately three cases per million people annually, predominantly in children and adolescents. In the past four decades, therapeutic approaches based on surgical resection combined with chemotherapy have played a major role in the treatment of osteosarcoma. Unfortunately, the overall survival rate for the disease remains unchanged, and the 5-year relative survival rate of patients presenting with localized disease is approximately 70%; however, for patients with lung metastasis, the survival rate is dramatically reduced to less than 30% [1–3]. Hence, a better understanding of osteosarcoma’s biological characteristics and molecular mechanisms is urgently needed to develop novel therapeutic strategies.

Extensive studies from our group and others have shown that CD151, the first member of the tetraspanin superfamily, contributes to the malignancy of human cancer [4–6]. CD151 is an established regulator of cell adhesion, migration, and metastasis and interacts with several partners, including MMPs (matrix metalloproteinases), cadherins, immunoglobulin proteins, and integrins, as well as other members of the tetraspanin family [7]. Our previous study demonstrated that CD151 positively correlates with lung metastasis and predicts poor prognosis in human osteosarcoma and that CD151 promotes osteosarcoma cell migration, invasion, and metastasis in vitro and in vivo [4, 8]. However, the exact mechanism underlying its contribution to tumorigenesis remains unclear.

Sphingolipids are important bioactive molecules involved in different and important biological processes in cells [9]. Sphingolipids are dysregulated in multiple cancers and are responsible for tumor growth, invasion, and metastasis through activation or inhibition of the cell signal transduction network [9, 10]. The sphingolipid network is complex, and sphingolipid abundance is highly regulated by various metabolic enzymes [9, 11, 12]. Serine palmitoyltransferase (SPT), the first rate-limiting enzyme for sphingolipid biosynthesis, consists mainly of long-chain base subunit 1 (SPTLC1 and 2/3) [13, 14]. Inhibition of sphingolipid biosynthesis can be toxic in different types of tumors, as has been shown with myriocin [15–18]. Myriocin, a specific inhibitor of SPT and sphingolipid synthesis, alone or in combination with chemotherapy, has produced encouraging results.

In this study, we demonstrated that CD151 reprograms sphingolipid metabolism that aids cell growth in osteosarcoma through global metabolomics, transcriptomics, and functional analysis in vitro and in vivo*.* Loss of CD151 impairs the activity of c-myc, resulting in decreased SPTLC1 expression and reprogramming of sphingolipid biosynthesis. Notably, CD151-overexpression in preclinical patient-derived osteosarcoma tumors were more vulnerable to the sphingolipid synthesis inhibitor, myriocin. Our work demonstrates that CD151 is a promising biomarker for predicting the effectiveness of sphingolipid metabolism inhibitors in osteosarcoma.

## Materials and methods

### Cell lines, antibodies, and reagents

Human osteosarcoma cell lines 143B, HOS-MNNG, and MG63 were purchased from the American Type Culture Collection (ATCC) and utilized according to the manufacturer’s instructions. ZOSM was obtained from the First Affiliated Hospital of Sun Yat-Sen University (Guangzhou, China). Mycoplasma contamination was excluded via PCR, and cell line identification was confirmed using short tandem repeat (STR) profiles. Osteosarcoma cells were cultured in DMEM (Corning, USA) containing 10% fetal bovine serum (FBS) and 1% penicillin/streptomycin. Myriocin and MG132 were purchased from MedChemExpress. BODIPY™ FL C_5_-Ceramide (D3521), BODIPY™ FL C5-Sphingomyelin (D3522), and Alexa Fluor 647-conjugated CTB (V34404) were purchased from Invitrogen (USA). The following antibodies were used: anti-β-actin (A5316) from Sigma-Aldrich and anti-CD151 (96262) and anti-c-myc (18583) from Cell Signaling Technology (USA). Anti-SPTLC1 (ab23287) antibody was purchased from Abcam (Cambridge, UK).

### CD151 knock-out cell lines

WT and CD151 KO osteosarcoma cells were generated using CRISPR/Cas9 mediated gene-editing technology. The lentivirus was produced by the co-transfection of lenti-CRISPR plasmids with pVSV-G and psPAX2 into HEK293T cells. HOS-MNNG and ZOSM cells were infected with lentiviral supernatants, which were selected in the presence of puromycin (Sigma-Aldrich) for one week. We further validated CD151 depletion by genomic DNA sequencing and western blot analysis. The sequences of CD151 sgRNAs are listed in Additional file 1: Table S1.

### Tissue samples

Human osteosarcoma tissues were obtained from patients at Shanghai General Hospital (China) during surgery. Appropriate consent was obtained from patients by the Shanghai General Hospital Ethics Committee before collecting specimens and patient information. Samples were then washed with ice-cold PBS (phosphate buffered saline) and stored frozen at – 80 °C before subsequent processing for RNA-seq.

### RNA-seq and quantitative real-time PCR

Total RNA was extracted from tissues and cell lines using TRIzol Reagent (Thermo), according to the manufacturer’s instructions. Subsequently, RNA samples were sent to Majorbio Technology Co., Ltd. (Shanghai, China) for high-throughput sequencing analysis. For quantitative real-time PCR, cDNA from isolated RNA was reverse-transcribed using the TaKaRa PrimeScript RT Reagent kit (TaKaRa, Japan) and then used for qPCR using SYBR Green-based system (Applied Biosystems) with gene-specific primers, and mRNA levels were normalized to GAPDH as a control. Primer sequences used are listed in Additional file 1: Table S1.

### Metabolic analysis

HOS-MNNG and ZOSM cells were extracted with cold methanol: water (4:1, v/v) (− 80 °C) to quantitatively analyze the metabolic profiles without any treatment. Samples were then submitted to Sensichip, Inc. (Shanghai, China), where the relative amounts of small molecular metabolites were determined using liquid chromatography coupled to mass spectrometry (LC/MS) platforms consisting of an electrospray ionization (ESI) source and a linear ion trap (LIT) mass analyzer.

### Luciferase reporter gene assay

The SPTLC1 promoter containing the wild-type c-myc binding sequences or the corresponding mutation in c-myc binding sequences was sub-cloned and inserted into the pGL3-basic vector, which was transfected into osteosarcoma cells. A Renilla luciferase control reporter plasmid was used to normalize the transfection efficiency. Luciferase reporter activity was assayed using the Dual-Luciferase Reporter Assay System (Promega, USA) according to the manufacturer’s instructions.

### ChIP-PCR

The chromatin immunoprecipitation (ChIP) assay was performed using anti- c-myc and a ChIP assay kit (Millipore, USA) according to the manufacturer’s instructions. Anti-IgG antibody was used as the negative control. Briefly, HOS-MNNG and ZOSM cells were fixed, harvested, and sonicated, and the samples immunoprecipitated with the respective antibodies. The bound DNA fragments were subjected to qPCR using four primer sets to amplify the c-myc-binding region(s) of the SPTLC1 promoter. Primers used to amplify specific genomic regions are listed in Additional file 1: Table S1.

### Cycloheximide (CHX) chase assay and Western blot

The osteosarcom cells HOS-MNNG, ZOSM, 143B, and MG63 were incubated in cycloheximide for the indicated times. The cells were then harvested and lysed for western blot analysis. Equal amounts of cell lysates were separated by SDS-PAGE and transferred onto the PVDF membranes (Millipore, USA). Following incubation with primary antibodies, the blots were incubated with secondary antibodies conjugated with HRP. Protein bands were visualized using Pierce ECL substrate (Thermo, USA). c-myc levels were measured with densitometric intensity using the ImageJ software.

### Ubiquitination assay

HOS-MNNG and ZOSM were transduced with vectors as indicated by the HA-tagged ubiquitin constructs. Before harvesting, the cells were incubated with MG132 to prevent proteasome-mediated protein degradation and with NP40-containing lysis buffer to prevent deubiquitination. Immunoprecipitation of the ubiquitinated c-myc protein was performed with an anti-c-myc antibody, followed by western blotting using an anti-HA antibody. In all experiments, equal amounts of HA-ubiquitin expression were verified by western blotting.

### Mouse xenograft experiments

All animal experiments were approved by the Shanghai General Hospital Animal Care and Use Committee. The tumor was established by injecting a cell suspension of (1 × 10^6^ HOS-MNNG cells) into a paraosseous site deep in the left caudal gastrocnemius of 6-week-old NOD/SCID nude mice [19, 20]. The mice were randomly divided into two groups for drug administration when the tumors reached 100–200 mm^3^ in volume (n = 5). Myriocin (0.5 mg/kg) or vehicle control was intraperitoneally injected into the mice every other day. For ethical reasons, the mice were euthanized when the largest tumor reached 2000 cm^3^ in size. Caliper measurements were used to assess tumor growth, and volumes were calculated using the formula V = 0.5 × L × (S)^2^ (L and S are the long and short diameters of the tumors, respectively.)

### PDX model

Patient-derived xenograft (PDX) murine models were generated using previously described methods [21]. To explain briefly, all patients had a confirmed pathological diagnosis of osteosarcoma at Shanghai General Hospital (Shanghai, China). Fresh tumor samples were collected immediately after surgery, cut into pieces, and engrafted into NOD-SCID/IL2Rγ^−/−^ (NSG) mice (Animal Resource Center, Shanghai, China) via subcutaneous transplantation. SA3831 (n = 11) and SA4009 (n = 7) PDX tumor-bearing mice treated with vehicle or myriocin. All the PDX studies complied with ethical regulations and were approved by the Shanghai General Hospital Animal Ethics Committee.

### Statistical analyses

Statistical analyses were performed using IBM SPSS 20 and GraphPad Prism v8. Data were presented as the mean ± SD (standard deviation), and each experiment was performed in triplicate. The unpaired Student’s t-test was used to compare the differences between the two groups. One-way analysis of variance (ANOVA) was used when more than two groups were involved. CD151 expression was positively and negatively correlated with the expression of other genes using Pearson’s correlation. The log-rank (Mantel-Cox) test was used to compare animal survival. Results with *P* < 0.05 were considered significant.

## Results

### CD151 regulates the sphingolipid metabolism pathway in osteosarcoma cells

**Fig. 1:**
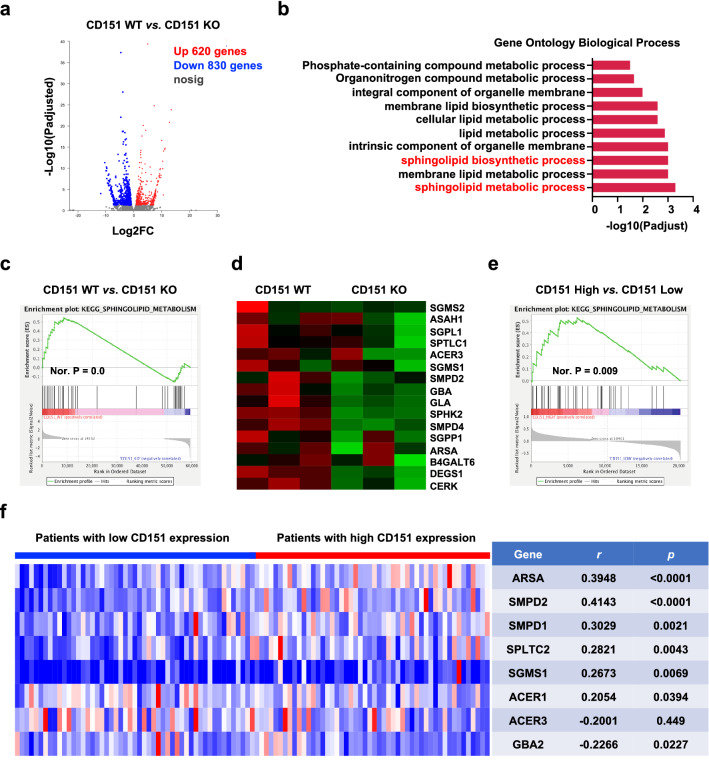
Transcriptic characterization of CD151 depletion in osteosarcoma and the clinical association between CD151 and sphingolipid metabolism. **a** Volcano plot of all genes from RNA-seq data in HOS-MNNG cells with CD151 WT compared with CD151 KO. The downregulated genes are shown in blue, and the upregulated genes are shown in red. **b** Geno Ontology (GO) enrichment analysis of differentially expressed genes between the WT and CD151 KO groups. GO terms comprising sphingolipid metabolic process and sphingolipid biosynthesis process are shown in red. **c** Gene Set Enrichment Analysis (GSEA) analysis identifies negatively enriched sphingolipid metabolism in HOS-MNNG cells after CD151 depletion. **d** Heatmap of the 16 genes contributing to sphingolipid metabolism GSEA enrichment plot. **e** Gene Set Enrichment Analysis (GSEA) analysis identifies positively enriched sphingolipid metabolism in patients samples with CD151 high expression. **f** The correlation between *CD151* mRNA expression and sphingolipid metabolism related-genes in 103 patients’ tumor samples

Our previously published study has shown that CD151 promotes osteosarcoma cell metastasis in vitro and in vivo [4]. To delineate the molecules and pathways responsible for the biological effects of CD151, global RNA sequencing (RNA-seq) was conducted in human osteosarcoma cells depleted of CD151 (CD151 KO) by CRISPR/CAS9 genome editing and compared to WT cells (Additional file 1: Fig. S1a). As shown in the volcano plot, RNA-seq analysis identified approximately 1450 differentially expressed genes (620 upregulated and 830 downregulated genes) upon CD151 silencing in HOS-MNNG cells (fold change > 1.5, FDR < 0.05) (Fig. 1a). The gene ontology (GO) enrichment analysis of the differentially expressed genes was mostly enriched in the sphingolipid metabolic process and sphingolipid biosynthetic process (Fig. 1b). Gene set enrichment (GSEA) confirmed that the expression of CD151 was positively correlated with the sphingolipid metabolism pathway (Fig. 1c). A heatmap of genes with significant changes in sphingolipid metabolism gene sets affected by CD151 KO is shown in Fig. 1d and Additional file 1: Fig. S1b. To further investigate the role of the sphingolipid pathway in osteosarcoma, we conducted a tissue microarray analysis of 103 clinical osteosarcoma specimens. The results showed that CD151 expression was significantly associated with the sphingolipid metabolic pathway (Fig. 1e, f). Data mining from the osteosarcoma GEO database (GSE42352, n = 127) was used to explore the CD151-related pathways and biological functions. The sphingolipid signaling pathway was consistently highly associated with CD151 expression (Additional file 1: Fig. S1c, d). These data suggested that CD151 regulated the sphingolipid metabolism pathway in osteosarcoma.

### CD151 regulates the sphingolipid intermediates in osteosarcoma cells

**Fig. 2:**
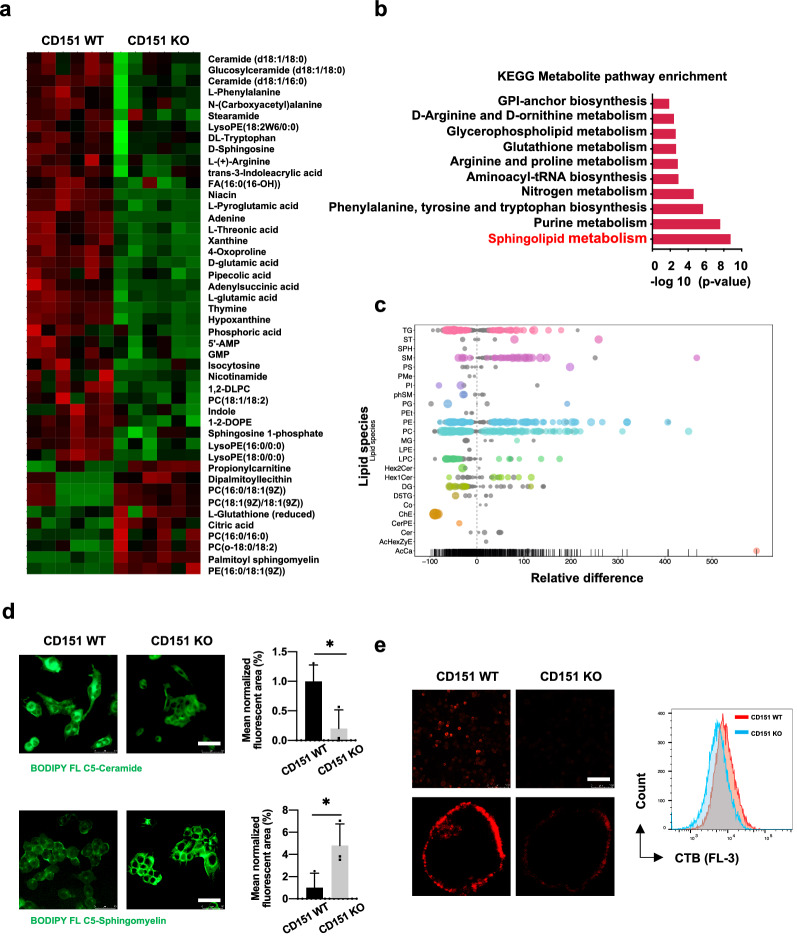
Metabolic characterization of CD151 depletion in osteosarcoma. **a** Liquid chromatography coupled to mass spectrometry (LC/MS) was used to measure the concentration of intermediates in HOS-MNNG cells. Heatmap showing significantly differently expressed metabolites altered by CD151 silencing. Shades of red and blue represent higher and lower levels of metabolites, respectively. **b** The top 10 enriched pathways from integrated pathway analysis of significantly changed metabolites. The red indicates the KEGG pathway related to sphingolipid metabolism. **c** Lipids were extracted from WT or CD151 KO HOS-MNNG cells and analyzed by LC/MS. Bubble plots represent the mean log_2_-transformed fold-change difference between cell lines. **d** Representative images and quantification of BODIPY FL-labeled ceramide or sphingomyelin was analyzed by confocal fluorescence imaging in HOS-MNNG cells. Scale bars: 50 μm. **e** HOS-MNNG cells with CD151 depletion were incubated with Alexa 555-conjugated CTB (red) to label GM1-containing lipid rafts. Cells were analyzed by confocal microscopy or flow cytometry. Scale bars: 50 μm. Comparisons were made using the two-tailed, unpaired Student’s t-test; *p < 0.05, **p < 0.01, ***p < 0.001

To investigate the metabolic vulnerability of cancer cells regulated by CD151, we conducted an untargeted metabolomic analysis in osteosarcoma cells using liquid chromatography coupled with mass spectrometry (LC/MS) analysis. The results of the heatmap analysis showed distinct clusters of metabolites in CD151 WT and KO cells, suggesting that their metabolic signatures were significantly different (Fig. 2a and Additional file 1: Fig. S2a). Pathway enrichment of these significantly changed metabolites further demonstrated that the sphingolipid metabolism pathway was most significantly altered in CD151 KO cells (Fig. 2b and Additional file 1: Fig. S2b). To study the abundance of sphingolipid metabolic intermediates, we performed mass spectrometry-based lipidomics analysis in CD151 depletion and control cells. As a result of CD151 depletion, the cellular lipid composition underwent dramatic changes, with significant alterations observed in sphingolipid components. Among sphingolipid species, significant decreases were observed for ceramide species upon depletion of CD151, while most sphingomyelin species were increased (Fig. 2c and Additional file 1: Fig. S2c).

To confirm whether CD151 regulates sphingolipid content, we loaded osteosarcoma cells with green fluorescent dye-labeled ceramide or sphingomyelin. Consistent with the observation in the metabolic analysis, a significant reduction of ceramide in CD151 KO cells was observed compared to WT cells, while accumulation of sphingomyelin in plasma membranes was observed (Fig. 2d and Additional file 1: Fig. S2d). Sphingolipids are structural molecules of cell lipid rafts on plasma membranes [9]. We next assessed whether CD151 affects the integrity of lipid rafts on cell surfaces. As expected, the staining of ganglioside GM1, a lipid raft marker, revealed less positive staining and lower mean fluorescence intensity in CD151 KO cells, which indicated that lipid raft formation on the plasma membrane was affected by CD151 (Fig. 2e and Additional file 1: Fig. S2e). Taken together, our transcriptomic and metabolomic measurements identified CD151 as a mediator of the reprogramming of sphingolipid metabolism in osteosarcoma cells.

### CD151 modulates sphingolipid metabolism by targeting SPTLC1

**Fig. 3:**
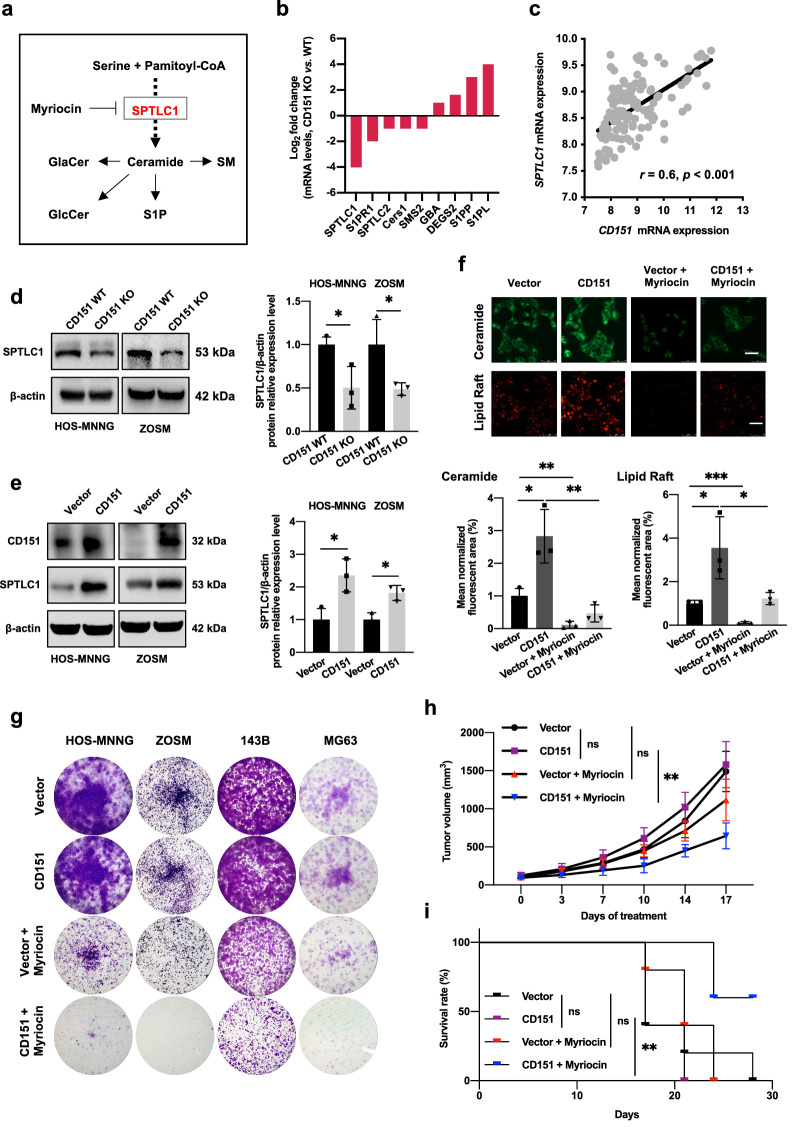
CD151 regulates sphingolipid metabolism in part through SPTLC1. **a** Diagram of sphingolipid metabolic pathway. **b** Expression of genes involved in sphingolipid metabolism was measured by PCR array in HOS-MNNG cells. Data are shown as log_2_-transformed fold change in CD151 silencing cells relative to control. **c** Spearman correlation analysis of *CD151* and *SPTLC1* mRNA levels in clinical osteosarcoma samples from the GEO database (GSE42352, n = 127). **d** and** e** Western blot analysis of SPTCL1 expression in cells with CD151 depletion (**d**) or overexpression (**e**). **f** The cellular levels of ceramide in WT and CD151 overexpression HOS-MNNG cells with or without myriocin treatment were analyzed by BODIPY FL-labeled ceramide confocal imaging (upper panel); The cellular levels of lipid rafts in WT and CD151 overexpression HOS-MNNG cells with or without myriocin treatment were analyzed by Alexa 555-conjugated CTB confocal imaging (low panel). Scale bars: 50 μm. **g** Representative images of clonogenic growth in CD151 overexpression cells treated with myriocin. **h** Tumor volume quantification of established tumors with CD151 overexpression treated with vehicle or myriocin (0.5 mg/kg). Data are presented as mean ± SD, n = 5. **i** Kaplan–Meier survival curve for each group. The survival rates were calculated when the last mouse was euthanized in the CD151 WT group. Comparisons were made using the two-tailed, unpaired Student’s t-test; The long-rank test was used to test for the significant differences in survival between the groups; *p < 0.05, **p < 0.01, ***p < 0.001

To identify the key enzymes involved in CD151-mediated sphingolipid metabolism reprogramming, we employed a qPCR-based custom array that included 80 crucial genes involved in sphingolipid metabolism and revealed that several related genes were dysregulated upon CD151 silencing (Fig. 3b and Additional file 1: Fig. S3a). Notably, the SPTLC1 mRNA levels in the control cells was substantially higher than that in the CD151 KO cells. SPTLC1 is the first rate-limiting enzyme in sphingolipid biosynthesis (Fig. 3a). Western blot analysis was used to examine the expression of SPTLC1 in osteosarcoma cells, and the results showed that the knockout of CD151 remarkably decreased, whereas CD151 overexpression significantly increased SPTLC1 protein levels (Fig. 3d, e and Additional file 1: Fig. S3b). We also examined the correlation between CD151 and SPTCL1 mRNA expression in a cohort of osteosarcoma GEO databases (GSE42352, n = 127). The results showed that CD151 and SPTLC1 expression levels were highly correlated in osteosarcoma samples (Fig. 3c). These data suggested that SPTLC1 played a critical role in CD151-mediated sphingolipid metabolism.

Next, we investigated whether CD151 mediates sphingolipid metabolism by SPTLC1 in osteosarcoma cells. CD151 overexpression and vector cells were treated with myriocin, a specific inhibitor of SPTLC1 and sphingolipid synthesis. Myriocin treatment significantly decreased ceramide accumulation and attenuated the positive staining and mean fluorescence intensity of the lipid rafts in CD151 overexpression cells (Fig. 3f and Additional file 1: Fig. S3d). Having established that CD151 reprograms sphingolipid metabolism primarily through SPTCL1, we further explored the possibility of developing new osteosarcoma treatments. The clonogenic growth of CD151-hyperactivated cells was significantly inhibited by myriocin, whereas control cells showed relatively weak responses (Fig. 3g). Furthermore, we examined the efficacy of myriocin in preventing CD151 high-expression tumor progression in vivo*.* Consistently, myriocin markedly reduced the tumor burden and extended survival time upon CD151 overexpression (Fig. 3h, i). Notably, xenograft models treated with myriocin did not show significant weight changes or obvious toxicity in the major organs (Additional file 1: Fig. S3e). Collectively, these data demonstrated that the sphingolipid metabolism pathway was important for tumorigenicity of CD151-overexpressing tumors.

### CD151 regulates the c-myc pathway in osteosarcoma

**Fig. 4:**
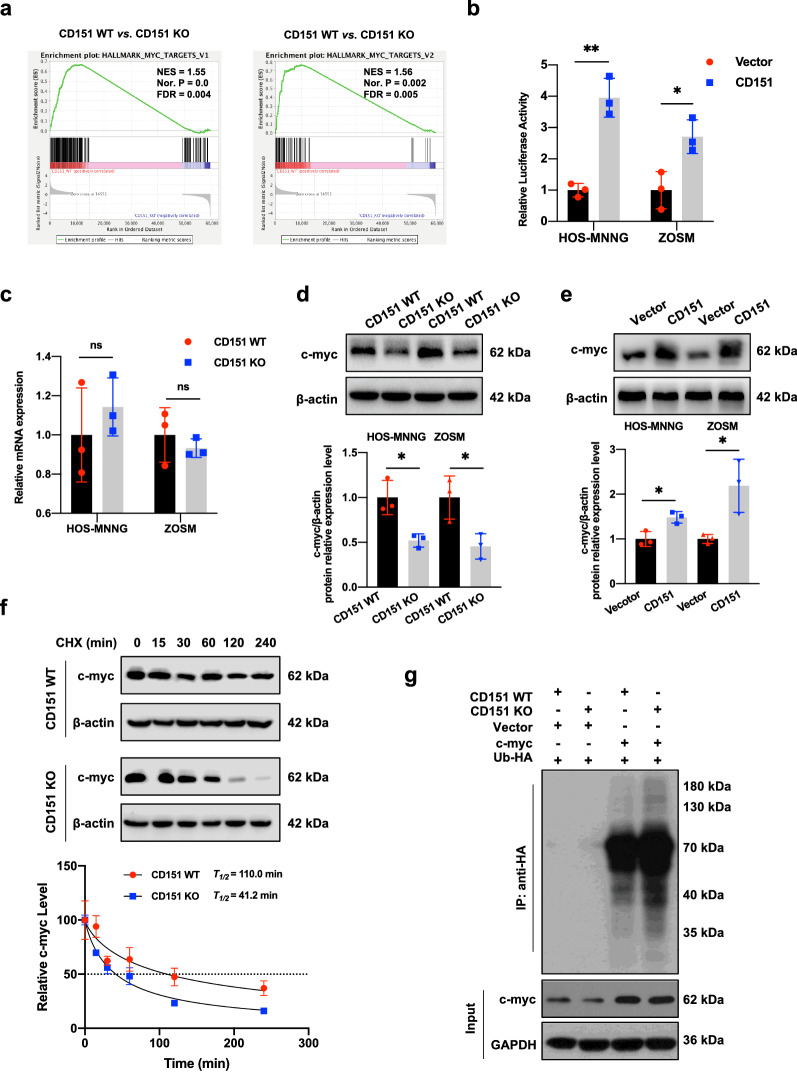
CD151 depletion enhances c-myc ubiquitination and subsequent degradation. **a** Gene set enrichment analysis (GSEA) based on the RNA-seq using Hallmarks gene sets between WT and CD151 KO HOS-MNNG cells. **b** Transcriptional activity of c-myc was measured using luciferase promoter-reporter assay in CD151 overexpression and vector cells. Data (mean ± SD) are presented as fold changes in normalized luciferase activity. **c** RT-qPCR analysis of *c-myc* expression in HOS-MNNG and ZOSM cells silenced for CD151 expression. **d** and** e** The c-myc expression was detected in HOS-MNNG and ZOSM cells after knockout (**d**) or overexpression (**e**) of CD151 by Western blot. **f** Time-course analysis of c-myc protein levels in CD151 depletion HOS-MNNG cells. c-myc band density relative to β-actin was quantified, and the ratio of c-myc protein/actin protein was artificially set as 1.0 for samples untreated with CHX to obtain half-time (T_1/2_) of c-myc. **g** c-myc ubiquitination was analyzed in CD151 depletion cells. HOS-MNNG cells were transfected with the indicated plasmids followed by treatment with MG132 for 6 h. Cell extracts were immunoprecipitated with an anti-HA antibody, and ubiquitination c-myc was detected by western blot. Comparisons were made using the two-tailed, unpaired Student’s t-test; *p < 0.05, **p < 0.01, ***p < 0.001

To decipher how CD151 controls cancer sphingolipid metabolism, we analyzed the correlation between key cancer metabolic regulators in cancer cells. Gene Set Enrichment Analysis (GSEA) demonstrated that CD151 depletion significantly affected the expression of c-myc targets (Fig. 4a and Additional file 1: Fig. S4a). We further analyzed the effects of CD151 on the c-myc pathway using a c-myc-responding promoter-reporter system. As shown in Fig. 4b, overexpression of CD151 significantly increased c-myc transcriptional activity in osteosarcoma cells. The c-myc mRNA and protein levels were examined in osteosarcoma to investigate whether CD151 activates endogenous c-myc. We observed that CD151 positively regulated c-myc protein expression in osteosarcoma cell lines without altering the mRNA level of c-myc (Fig. 4c–e, and Additional file 1: Fig. S4b), suggesting that the regulation of c-myc by CD151 occurs at the post-transcriptional level. To examine whether CD151 promotes c-myc stabilization in osteosarcoma cells, we measured the half-life of c-myc in the presence and absence of CD151 using a cycloheximide chase assay. As shown in Fig. 4f and Additional file 1: Fig. S4c, depletion of CD151 significantly shortened the half-life of endogenous c-myc in osteosarcoma cells. The ubiquitin-dependent proteasome pathway is thought to be responsible for the rapid c-myc protein turnover. To investigate whether c-myc polyubiquitination was induced by CD151 depletion, osteosarcoma cells were transfected with a recombinant plasmid encoding HA-tagged ubiquitin. Compared to WT cells, CD151 KO cells had significantly higher amounts of polyubiquitinated c-myc (Fig. 4g and Additional file 1: Fig. S4d). Our data demonstrated that CD151 depletion decreased c-myc expression because of the increase in c-myc ubiquitination for protein degradation.

### CD151 activates transcription of SPTLC1 through c-myc in osteosarcoma

**Fig. 5:**
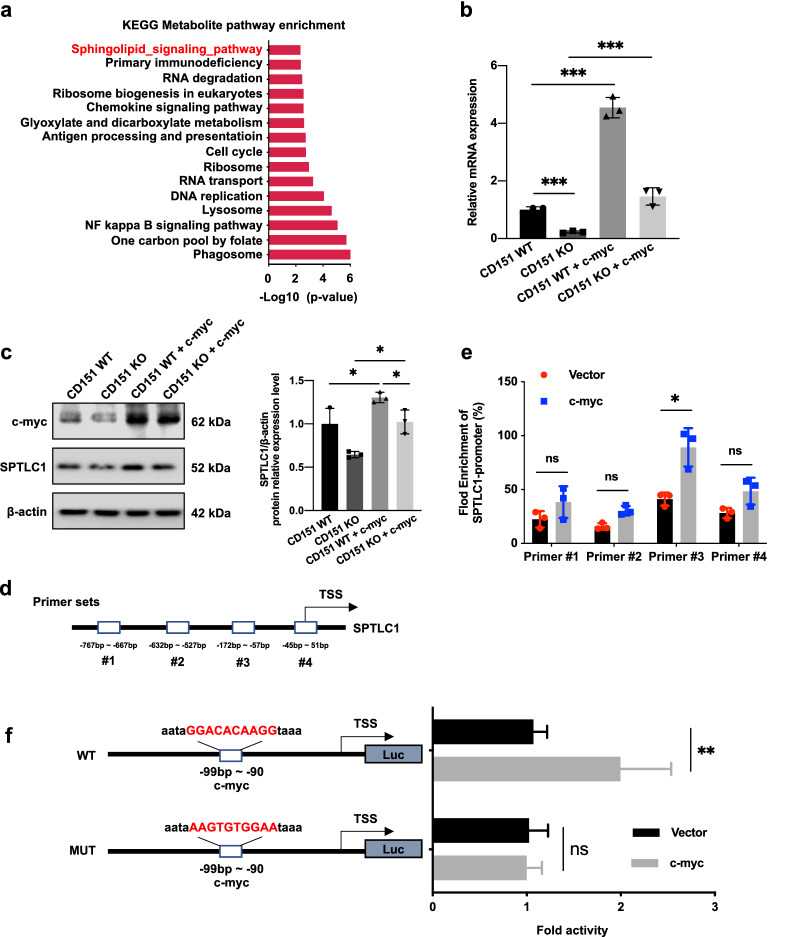
CD151 regulates the transcription of SPTLC1 by c-myc. **a** KEGG analysis for c-myc-related differentially expressed genes based on GEO database (GSE42352, n = 127). The red indicates the KEGG pathway related to sphingolipid metabolism. **b** and **c** *SPTLC1* mRNA and protein levels in CD151 depletion cells stably infected with vector or c-myc-expressing lentiviruses were analyzed by RT-qPCR (**b**) and Western blot (**c**). Data are shown as mean ± SD of triplicate experiments. **d** and** e** Schematic representation of the promoter region in the human *SPTLC1* gene (**d**). ChIP analysis from HOS-MNNG cells was performed with control IgG or c-myc antibody as indicated. The presence of the *SPTLC1* binding was detected by qPCR using primers in either conver c-myc binding region (#3) or the negative unrelated region (#1, #2, and #4). Quantification of enrichments is represented as fold-enrichment over lgG control. **f** HOS-MNNG cells were transfected with an empty vector or c-myc plus the wild-type *SPTLC1* promoter (WT) or mutated promoter (MUT) for measuring luciferase activity. Data are the mean ± SD, and the data are representative of three independent experiments

c-myc, the major oncogenic transcription factor, has been implicated in various cellular metabolic processes, including sphingolipid metabolism [22, 23]. We searched the GEO database (GSE42352, n = 127) using the Kyoto Encyclopedia of Genes and Genomes (KEGG) pathway enrichment analysis and found that c-myc expression was closely associated with the sphingolipid signaling pathway in osteosarcoma (Fig. 5a). It was speculated that CD151 might interact with c-myc pathways and regulate sphingolipid metabolism key enzyme SPTLC1 expression in osteosarcoma cells. SPTLC1 expression was examined in cells transfected with a c-myc lentiviral overexpression vector into CD151-depletion cells to verify this hypothesis. The results suggested that the reduction in *SPTLC1* mRNA and protein levels by CD151 silencing was partially rescued by overexpression of c-myc (Fig. 5b, c, and Additional file 1: Fig. S5a, b). Next, we explored whether c-myc regulates SPTLC1 at the transcriptional level. We performed chromatin immunoprecipitation (ChIP) assays using four pairs of primers to survey the putative binding site(s) within the SPTLC1 promoter region (Fig. 5d and Additional file 1: Fig. S5c). c-myc exhibited strong binding to SPTLC1 promoter primer set #3, but showed relatively weak binding to other primer sets (Fig. 5e and Additional file 1: Fig. S5d). To further evaluate whether promoter primer set #3 conferred c-myc -dependent transcriptional activity, we constructed an *SPTLC1* promoter vector (WT) and a site-mutated mutant (MUT) based on a luciferase reporter gene plasmid. We found that *SPTLC1* promoter activities from WT were greatly increased, whereas no obvious changes were detected from MUT in osteosarcoma cells overexpressing c-myc compared to the empty vector (Fig. 5f and Additional file 1: Fig. S5e), respectively. These results indicated that CD151 activated SPTLC1 transcription through c-myc in osteosarcoma.

### Sphingolipid synthesis inhibitor myriocin selectively suppresses the growth of CD151-overexpression tumors in the PDX model

**Fig. 6:**
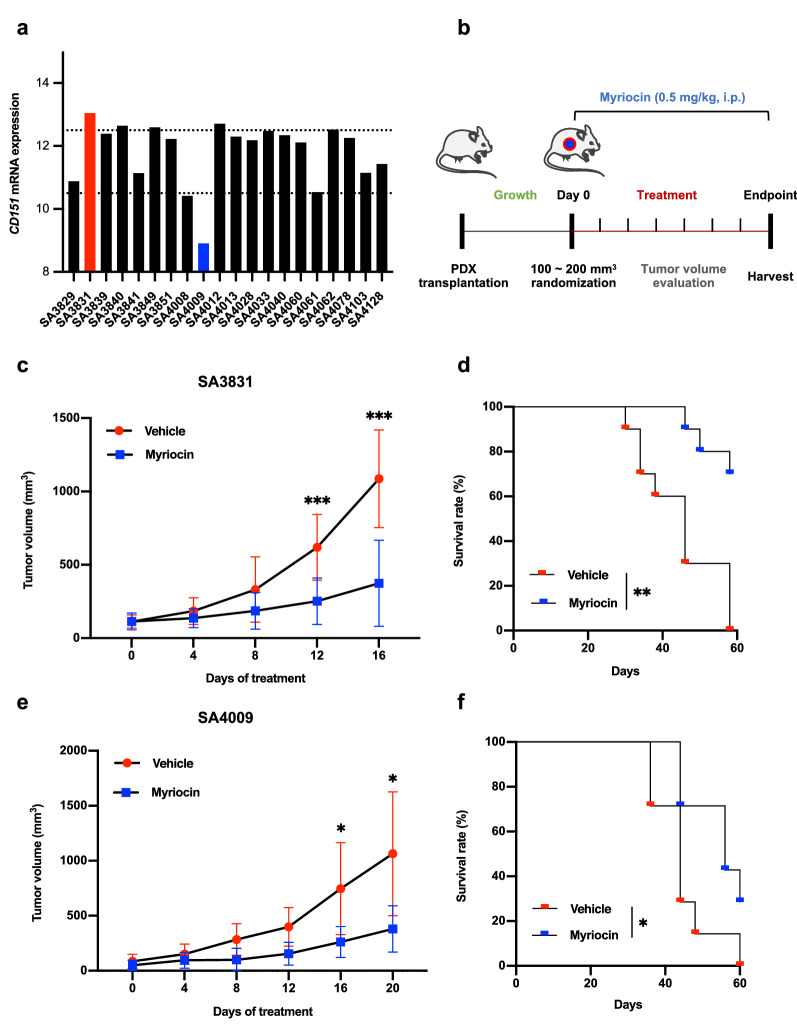
Myriocin selectively suppresses the growth of CD151-overexpression tumors in the PDX model. **a** *CD151* mRNA expression levels in several osteosarcoma PDX model cases were evaluated by RNA-seq. Columns of red and blue represent high and low levels of CD151 expression, respectively. **b** Schematic of treatment strategy with myriocin in PDX models. **c–f** Tumor volume quantification (**c**, **e**) and Kaplan–Meier survival curve (**d**, **f**) of SA3831 (n = 11) and SA4009 (n = 7) PDX tumor-bearing mice treated with vehicle or myriocin. Comparisons were made using the two-tailed, unpaired Student’s t-test; The long-rank test was used to test for the significant differences in survival between the groups; *p < 0.05, **p < 0.01, ***p < 0.001

Based on the finding that CD151 is a key regulator of sphingolipid metabolism, we conducted an initial translational experiment in osteosarcoma. To test the efficacy of the sphingolipid synthesis inhibitor, myriocin, in preventing CD151-induced tumor progression, we utilized PDX murine models, which mimic primary tumor heterogeneity and histopathology. To evaluate the therapeutic potential of targeting CD151 in vivo, we first detected *CD151* mRNA expression levels in several osteosarcoma PDX models (Fig. 6a). Based on the expression of CD151 in the primary tumor, one high expression case, SA3831, and one low expression case, SA4008, were chosen for further study. When implanted PDX mammary tumor volume reached approximately 100–200 mm^3^, mice were randomly grouped and treated intraperitoneally with myriocin (0.5 mg/kg) or vehicle control every other day (Fig. 6b). In a high expression model of CD151, myriocin treatment potently suppressed the growth of established tumors and prolonged the survival of tumor-bearing mice (Fig. 6c, d). However, its effectiveness was reduced in models with low CD151 expression (Fig. 6e, f). Ki67 staining results also strongly support our conclusion (Additional file 1: Fig. S6a). Collectively, these data demonstrated the in vivo efficacy of myriocin in inhibiting osteosarcoma growth and indicated that CD151 expression was a potentially useful biomarker for predicting the efficacy of sphingolipid metabolism inhibition.

## Discussion

Investigating the biological characteristics and molecular mechanisms of osteosarcoma is important for developing new therapies [24]. Previous studies have suggested an association between CD151 and distinct consequences in osteosarcoma development [4]. However, the potential of CD151 as a therapeutic target has not yet been sufficiently explored. In this study, we conducted an integrated transcriptomic and metabolomic analysis of osteosarcoma and identified sphingolipid metabolism as the top CD151-regulated pathway. While searching for a potential mechanism for CD151-mediated sphingolipid metabolism, we found that CD151 depletion can downregulate c-myc expression by enhancing c-myc polyubiquitination. We further proved that c-myc transactivates SPTCL1, the first rate-limiting enzyme in sphingolipid synthesis. More importantly, we demonstrated that patients with high CD151 levels might benefit more from sphingolipid metabolism inhibition, according to the results from the PDX and genetically engineered mouse models.

To our knowledge, the present study is the first to demonstrate that the loss of CD151 links altered cancer genomes with sphingolipid metabolism reprogramming to drive tumor growth in osteosarcoma. Sphingolipids, including the bioactive lipids ceramide and sphingomyelin, have been implicated in the regulation of tumorigenic capacity [9]. For instance, ceramide is an inhibitory lipid in regulatory mechanisms of PI3K-controlled motility [25]; Furthermore, plasma membrane ceramide was an acute signaling effector necessary and sufficient for regulation of cell adhesion and cell migration under chemotherapeutical stress [26]. We showed that CD151 silencing significantly regulated the expression of sphingolipid metabolism-related enzymes, which play key roles in the induction of cancer cell adhesion and metastasis. CD151 regulates sphingolipid metabolism primarily through SPTCL1 in osteosarcoma. SPT deficiency can impair adherens junctions and promote tumorigenesis, and sphingomyelin supplementation can partially correct the phenotype mediated by SPT deficiency [27].

Importantly, the metabolic phenotype reprogrammed by CD151 could further serve as a point of intervention and biomarker for osteosarcoma progression and therapeutic susceptibility. We conducted preliminary osteosarcoma translational experiments in light of our finding that CD151 was a key mediator of sphingolipid metabolism. Notably, we found that patients with CD151 overexpression might benefit more from inhibition of sphingolipid metabolism. A stronger conclusion was reached based on the preliminary positive results obtained from the PDX model, which replicated clinical conditions more closely [28]. CD151 has been recognized as a prognostic marker and therapeutic target for osteosarcoma but has been less explored for direct targeting of molecular inhibitors [29]. The application of sphingolipid metabolism pathway inhibitors will provide new insights into the development of novel CD151-based targeted therapies for osteosarcoma.

Furthermore, it is worth noting that our study indicates that CD151 regulates sphingolipid metabolism in part through a c-myc -mediated expression of SPTLC1. This is significant as aberrantly high expression of c-myc is a common basis for tumorigenesis in osteosarcoma, and c-myc has also been recognized as an important transcriptional regulator of cell metabolism processes, including sphingolipid metabolism [30–32]. In the current study, we demonstrated that CD151 inhibited the polyubiquitination of c-myc, thereby increasing c-myc protein stability. Studies from our group and others have demonstrated that CD151 regulates the ERK and GSK-3β signaling pathways in multiple types of malignant tumors [33, 34]. The ERK and GSK-3β signaling pathways phosphorylate and stabilize c-myc by regulating its ubiquitin-mediated degradation [30]. Future studies are required to explore whether these signaling pathways in osteosarcoma cells regulate c-myc expression in response to CD151 challenge.

The present study indicates that CD151 is critically important for regulating sphingolipid metabolism in osteosarcoma. CD151 depletion regulated the transcription of SPTLC1 enzymes by enhancing c-myc polyubiquitination and subsequent degradation. Importantly, inhibiting the CD151/sphingolipid metabolism pathway would greatly inhibit osteosarcoma tumorigenicity, and CD151 expression is a potentially useful biomarker for predicting the efficacy of sphingolipid metabolism inhibition. Further studies need to be undertaken to determine the clinical relevance of these findings.

## Supplementary Information

**Additional file 1: Figure S1. a.** Western blot demonstrating CRISPR/CAS9-mediated CD151 knockout in indicated cells. **b.** Heatmap of the sphingolipid metabolism genes regulated by CD151 depletion in ZOSM cells from RNA-seq. **c.** Kyoto Encyclopedia of Genes and Genomes (KEGG) analysis of transcriptomic profiles of osteosarcoma patients based on the GEO database (GSE42352, n = 127). The red indicates the KEGG pathway related to sphingolipid metabolism. **d**. Heatmap of osteosarcoma patients showed changes of sphingolipid metabolism genes that express a high level of CD151 compared to tumors with low CD151 expression. **Figure S2. a.** Liquid chromatography coupled to mass spectrometry (LC/MS) was used to measure the concentrations of intermediates in ZOSM cells. Heatmap showing significantly differently expression metabolites altered by CD151 silencing. Shades of red and blue represent higher and lower levels of metabolites, respectively. **b.** The top 10 enriched pathways from integrated pathway analysis of significantly changed metabolites. The red indicates the KEGG pathway related to sphingolipid metabolism. **c**. Lipids were extracted from WT or CD151 KO ZOSM cells and analyzed by LC/MS. Bubble plots represent the mean log_2_-transformed fold-change difference between cell lines. **d.** The intensity of BODIPY FL-labeled ceramide or sphingomyelin was analyzed by confocal fluorescence imaging in ZOSM cells. Scale bars: 50 μm. **e**. ZOSM cells with CD151 depletion were incubated with Alexa 555-conjugated CTB (red) to label GM1-containing lipid rafts. Cells were analyzed by confocal microscopy or flow cytometry. Scale bars: 50 μm. **Figure S3. a.** Expression of genes involved in sphingolipid metabolism was measured by PCR array in ZOSM cells. Data are shown as log_2_-transformed fold change in CD151 silencing cells relative to control. **b.** Western blot analysis of SPTCL1 expression in cells with CD151 overexpression in indicated cells. **c.** The cellular levels of ceramide in vector and CD151 overexpression ZOSM cells with or without myriocin treatment were analyzed by BODIPY FL-labeled ceramide confocal imaging. Scale bars: 50 μm. **d**. The cellular levels of lipid rafts in vector and CD151 overexpression ZOSM cells with or without myriocin treatment were analyzed by Alexa 555-conjugated CTB confocal imaging. Scale bars: 50 μm. **e.** Mice weight quantification of established tumors with CD151 overexpression treated with vehicle or myriocin (0.5 mg/kg). Data are presented as mean ± SD, n = 5. **Figure S4. a**. Gene set enrichment analysis (GSEA) based on the RNA-seq using Hallmarks gene sets between WT and CD151 KO ZOSM cells. **b**. The c-myc expression was detected in the indicated cells with CD151 overexpression by western blot. **c.** Time-course analysis of c-myc protein levels in CD151 depletion ZOSM cells. c-myc band density relative to β-actin was quantified, and the ratio of c-myc protein/actin protein was artificially set as 1.0 for samples untreated with CHX to obtain half-time (T_1/2_) of c-myc. **d.** c-myc ubiquitination was analyzed in CD151 depletion cells. ZOSM cells were transfected with the indicated plasmids followed by treatment with MG132 for 6 h. Cell extracts were immunoprecipitated with an anti-HA antibody, and ubiquitination c-myc was detected by western blot. **Figure S5. a-b.** *SPTLC1* mRNA and protein levels in CD151 depletion cells stably infected with vector or c-myc-expressing lentiviruses were analyzed by RT-qPCR (A) and Western blot (B). Data are shown as mean ± SD of triplicate experiments. **c-d.** Schematic representation of the promoter region in the human *SPTLC1* gene (C). ChIP from ZOSM cells was performed with control IgG or c-myc antibody as indicated. The presence of the *SPTLC1* binding sites was detected by qPCR using primers. Quantification of enrichments is represented as fold-enrichment over lgG control. **e.** ZOSM cells were transfected with an empty vector or c-myc plus the wild-type *SPTLC1* promoter (WT) or mutated promoter (MUT) for measuring luciferase activity. Data are the mean ± SD, and the data are representative of three independent experiments. **Figure S6. a.** The representative images of Ki67 staining from SA3831 and SA4009 PDX tumor-bearing mice treated with vehicle or myriocin. Scale bars: 100 μm. **Table S1.**

## Data Availability

The datasets obtained and analyzed for this study will be available from the corresponding author at a reasonable request.
