# Supplementary material for: Inhibition of sphingolipid metabolism in osteosarcoma protects against CD151-mediated tumorigenicity
Source: Cell Biosci. 2022 Oct 8;12:169. doi: 10.1186/s13578-022-00900-9 (PMC9548188; doi:10.1186/s13578-022-00900-9)
Supplement: Supplementary file 1 — Additional file 1: Figure S1. a. Western blot demonstrating CRISPR/CAS9-mediated CD151 knockout in indicated cells. b. Heatmap of the sphingolipid metabolism genes regulated by CD151 depletion in ZOSM cells from RNA-seq. c. Kyoto Encyclopedia of Genes and Genomes (KEGG) analysis of transcriptomic profiles of osteosarcoma patients based on the GEO database (GSE42352, n = 127). The red indicates the KEGG pathway related to sphingolipid metabolism. d. Heatmap of osteosarcoma patients showed changes of sphingolipid metabolism genes that express a high level of CD151 compared to tumors with low CD151 expression. Figure S2. a. Liquid chromatography coupled to mass spectrometry (LC/MS) was used to measure the concentrations of intermediates in ZOSM cells. Heatmap showing significantly differently expression metabolites altered by CD151 silencing. Shades of red and blue represent higher and lower levels of metabolites, respectively. b. The top 10 enriched pathways from integrated pathway analysis of significantly changed metabolites. The red indicates the KEGG pathway related to sphingolipid metabolism. c. Lipids were extracted from WT or CD151 KO ZOSM cells and analyzed by LC/MS. Bubble plots represent the mean log2-transformed fold-change difference between cell lines. d. The intensity of BODIPY FL-labeled ceramide or sphingomyelin was analyzed by confocal fluorescence imaging in ZOSM cells. Scale bars: 50 μm. e. ZOSM cells with CD151 depletion were incubated with Alexa 555-conjugated CTB (red) to label GM1-containing lipid rafts. Cells were analyzed by confocal microscopy or flow cytometry. Scale bars: 50 μm. Figure S3. a. Expression of genes involved in sphingolipid metabolism was measured by PCR array in ZOSM cells. Data are shown as log2-transformed fold change in CD151 silencing cells relative to control. b. Western blot analysis of SPTCL1 expression in cells with CD151 overexpression in indicated cells. c. The cellular levels of ceramide in vector and CD151 overexpr [file 13578_2022_900_MOESM1_ESM.docx]

Supplementary Materials for

Inhibition of sphingolipid metabolism in osteosarcoma protects from CD151-mediated tumorigenicity

Hongsheng Wang, Xinmeng Jin, Yangfeng Zhang, Zhuoying Wang, Tao Zhang, Jing Xu, Jiakang Shen, Pengfei Zan, Mengxiong Sun, Chongren Wang, Yingqi Hua, Xiaojun Ma, Wei Sun

**Correspondence to**: viv-sun@sjtu.edu.cn, ma-xiaojun@foxmail.com, or yhua@shsmu.edu.cn.

**a.** Western blot demonstrating CRISPR/CAS9-mediated CD151 knockout in indicated cells. **b.** Heatmap of the sphingolipid metabolism genes regulated by CD151 depletion in ZOSM cells from RNA-seq. **c.** Kyoto Encyclopedia of Genes and Genomes (KEGG) analysis of transcriptomic profiles of osteosarcoma patients based on the GEO database (GSE42352, n=127). The red indicates the KEGG pathway related to sphingolipid metabolism. **d**. Heatmap of osteosarcoma patients showed changes of sphingolipid metabolism genes that express a high level of CD151 compared to tumors with low CD151 expression.

**a.** Liquid chromatography coupled to mass spectrometry (LC/MS) was used to measure the concentrations of intermediates in ZOSM cells. Heatmap showing significantly differently expression metabolites altered by CD151 silencing. Shades of red and blue represent higher and lower levels of metabolites, respectively. **b.** The top 10 enriched pathways from integrated pathway analysis of significantly changed metabolites. The red indicates the KEGG pathway related to sphingolipid metabolism. **c**. Lipids were extracted from WT or CD151 KO ZOSM cells and analyzed by LC/MS. Bubble plots represent the mean log_2_-transformed fold-change difference between cell lines. **d.** The intensity of BODIPY FL-labeled ceramide or sphingomyelin was analyzed by confocal fluorescence imaging in ZOSM cells. Scale bars: 50μm. **e**. ZOSM cells with CD151 depletion were incubated with Alexa 555-conjugated CTB (red) to label GM1-containing lipid rafts. Cells were analyzed by confocal microscopy or flow cytometry. Scale bars: 50 μm.

**a.** Expression of genes involved in sphingolipid metabolism was measured by PCR array in ZOSM cells. Data are shown as log_2_-transformed fold change in CD151 silencing cells relative to control. **b.** Western blot analysis of SPTCL1 expression in cells with CD151 overexpression in indicated cells. **c.** The cellular levels of ceramide in vector and CD151 overexpression ZOSM cells with or without myriocin treatment were analyzed by BODIPY FL-labeled ceramide confocal imaging. Scale bars: 50 μm. **d**. The cellular levels of lipid rafts in vector and CD151 overexpression ZOSM cells with or without myriocin treatment were analyzed by Alexa 555-conjugated CTB confocal imaging. Scale bars: 50 μm. **e.** Mice weight quantification of established tumors with CD151 overexpression treated with vehicle or myriocin (0.5 mg/kg). Data are presented as mean ± SD, n = 5.

**a**. Gene set enrichment analysis (GSEA) based on the RNA-seq using Hallmarks gene sets between WT and CD151 KO ZOSM cells. **b**. The c-myc expression was detected in the indicated cells with CD151 overexpression by western blot. **c.** Time-course analysis of c-myc protein levels in CD151 depletion ZOSM cells. c-myc band density relative to β-actin was quantified, and the ratio of c-myc protein/actin protein was artificially set as 1.0 for samples untreated with CHX to obtain half-time (T_1/2_) of c-myc. **d.** c-myc ubiquitination was analyzed in CD151 depletion cells. ZOSM cells were transfected with the indicated plasmids followed by treatment with MG132 for 6 h. Cell extracts were immunoprecipitated with an anti-HA antibody, and ubiquitination c-myc was detected by western blot.

**a-b.** *SPTLC1* mRNA and protein levels in CD151 depletion cells stably infected with vector or c-myc-expressing lentiviruses were analyzed by RT-qPCR (A) and Western blot (B). Data are shown as mean ± SD of triplicate experiments. **c-d.** Schematic representation of the promoter region in the human *SPTLC1* gene (C). ChIP from ZOSM cells was performed with control IgG or c-myc antibody as indicated. The presence of the *SPTLC1* binding sites was detected by qPCR using primers. Quantification of enrichments is represented as fold-enrichment over lgG control. **e.** ZOSM cells were transfected with an empty vector or c-myc plus the wild-type *SPTLC1* promoter (WT) or mutated promoter (MUT) for measuring luciferase activity. Data are the mean ± SD, and the data are representative of three independent experiments.

**a.** The representative images of Ki67 staining from SA3831 and SA4009 PDX tumor-bearing mice treated with vehicle or myriocin. Scale bars: 100 μm.

Supplementary Table 1.

| **Nmae** | **Application** | **Sequence** |
| --- | --- | --- |
| sgRNA-CD151-F | CIRSPR-CAS | ACCGTACCTACAATTGCTGCTTC |
| sgRNA-CD151-R | CIRSPR-CAS | AAACGAAGCAGCAATTGTAGGTA |
| PCR-c-myc-F | qRT-PCR | GGCTCCTGGCAAAAGGTCA |
| PCR-c-myc-R | qRT-PCR | CTGCGTAGTTGTGCTGATGT |
| PCR-SPTC1-F | qRT-PCR | GCAGTGTTGAAGGAAAAGTGCGG |
| PCR-SPTC1-R | qRT-PCR | CAGTGCTCTCTTCCAGTTGTAGG |
| PCR-GAPDH-F | qRT-PCR | GGAGCGAGATCCCTCCAAAAT |
| PCR-GAPDH-R | qRT-PCR | GGCTGTTGTCATACTTCTCATGG |
| ChIP-SPTLC1-P-F1 | qRT-PCR for ChIP | AAAGTCTTGTCCCTATGATCTCAA |
| ChIP-SPTLC1-P-R1 | qRT-PCR for ChIP | AGAACCTTTTAGAGCCTTCCAA |
| ChIP-SPTLC1-P-F2 | qRT-PCR for ChIP | GCAAAGCCTTGGCCTATGTC |
| ChIP-SPTLC1-P-R2 | qRT-PCR for ChIP | AAGTAATCCAGCAATGCATTATCA |
| ChIP-SPTLC1-P-F3 | qRT-PCR for ChIP | CCCAGCTGATGACCAAGTGT |
| ChIP-SPTLC1-P-R3 | qRT-PCR for ChIP | GTGGGATGCCGCAGTTTTTC |
| ChIP-SPTLC1-P-F4 | qRT-PCR for ChIP | CAGCCTCCTGTCAACCAGTG |
| ChIP-SPTLC1-P-R4 | qRT-PCR for ChIP | GTCGCTGCTCAGTGTCGTTA |
